# Blood flow controls coagulation onset via the positive feedback of factor VII activation by factor Xa

**DOI:** 10.1186/1752-0509-4-5

**Published:** 2010-01-26

**Authors:** Alexey M Shibeko, Ekaterina S Lobanova, Mikhail A Panteleev, Fazoil I Ataullakhanov

**Affiliations:** 1 National Research Center for Hematology, 4a Novyi Zykovskii pr., Moscow 125167, Russia; 2 Albert Eye Research Institute, Duke University Medical Center, Durham, North Carolina 27710, USA; 3 Center for Theoretical Problems of Physicochemical Pharmacology, 4 Kosygina str., Moscow 119991, Russia; 4 Department of Physics, Moscow State University, 1 Vorobyevy gory, Moscow 119991, Russia

## Abstract

**Background:**

Blood coagulation is a complex network of biochemical reactions, which is peculiar in that it is time- and space-dependent, and has to function in the presence of rapid flow. Recent experimental reports suggest that flow plays a significant role in its regulation. The objective of this study was to use systems biology techniques to investigate this regulation and to identify mechanisms creating a flow-dependent switch in the coagulation onset.

**Results:**

Using a detailed mechanism-driven model of tissue factor (TF)-initiated thrombus formation in a two-dimensional channel we demonstrate that blood flow can regulate clotting onset in the model in a threshold-like manner, in agreement with existing experimental evidence. Sensitivity analysis reveals that this is achieved due to a combination of the positive feedback of TF-bound factor VII activation by activated factor X (Xa) and effective removal of factor Xa by flow from the activating patch depriving the feedback of "ignition". The level of this trigger (i.e. coagulation sensitivity to flow) is controlled by the activity of tissue factor pathway inhibitor.

**Conclusions:**

This mechanism explains the difference between red and white thrombi observed *in vivo *at different shear rates. It can be speculated that this is a special switch protecting vascular system from uncontrolled formation and spreading of active coagulation factors in vessels with rapidly flowing blood.

## Background

Blood coagulation is a complex reaction network that functions to form a fibrin clot that covers damaged vessel wall and prevents blood loss [1]. The clotting process is initiated by tissue factor (TF), a transmembrane protein exposed in the damaged parts of the wall. This protein forms a complex called extrinsic tenase with plasma protein activated factor VII (VIIa). Extrinsic tenase activates factor X, which activates thrombin, the main protein of blood coagulation. Activated factor X (factor Xa) activates factor VII in complex VII-TF (inactive extrinsic tenase), thus forming a positive feedback. Extrinsic tenase is inhibited by tissue factor pathway inhibitor (TFPI) in a complex factor Xa-dependent manner [2]. Thrombin forms fibrin, which polymerizes to create a clot.

Although reactions of the coagulation cascade are well known, and no new essential components of this system have been discovered over the last fifteen years [1], the present understanding of the functioning of this system is limited. The incredible biochemical complexity of coagulation additionally complicated by protein diffusion and blood flow makes it extremely difficult to establish a correlation between the roles of individual reactions and the functioning of the clotting system in vivo as a whole. For diffusion, a number of recent studies brought attention to the essential role of spatial non-uniformity and rate-limiting diffusion of specific coagulation factors thus proposing new concepts of clotting regulation, alternative to the classic "cascade" paradigm [1,3-6]. This is not the case for blood flow.

A major role of flow in hemostasis and thrombosis was recognized as early as 19th century, flow being one of the components of the famous Virchow's triad [7]. Two primary hemostatic mechanisms, platelet plug formation and blood coagulation, are known to differently depend on the flow conditions: platelet adhesion and aggregation require high blood flow velocities, while fibrin deposition occurs better in slowly flowing blood [8,9]. Moreover, a recent report suggests that fibrin clot formation is inhibited by flow in a threshold-like manner [10]. This can be illustrated *in vivo *by formation of fibrin-rich red thrombi containing erythrocytes in veins (where shear rate is low) and of platelet-rich white thrombi in arteries [11]. However, the phenomenon of blood clotting inhibition by flow has not been studied in detail and was assumed to be a self-evident consequence of the removal of active coagulation factors from the site of vascular damage by flow.

In order to gain insight into this phenomenon, a modular decomposition strategy was used. We created a detailed quantitative mechanism-driven mathematical model of thrombus formation in flowing plasma. In agreement with experimental reports, clot formation in the model depended on blood flow shear rate in a threshold-like manner. Sensitivity analysis of this model was performed to identify reactions forming a module (or subsystem) within this biochemical network that was responsible for this effect. We demonstrated that a specially designed positive feedback of factor VII activation combined with chemical inhibition of extrinsic tenase and flow-induced removal of factor Xa becomes a "switcher" that can "decide": whether to start clotting or to abstain from it. It can be speculated that this switching can serve to prevent uncontrolled spreading of coagulation factor by flow throughout the vasculature.

## Results

### The effect of flow on fibrin thrombus formation

**Figure 1:**
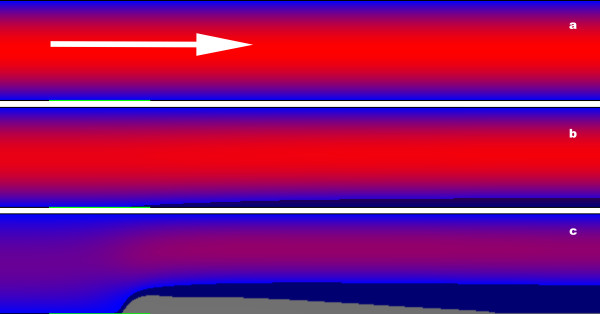
**Fibrin clot in the presence of flow**. Computer simulations were performed as described under the Methods section. Size of simulation area 1000 μm × 6000 μm; activator size 1000 μm. The time is 0 min **(a)**, 15 min **(b) **and 25 min **(c) **since the beginning of coagulation, wall shear rate is 17 s^-1^. Arrow shows the direction of flow. Blue-red gradient shows the value of horizontal component of flow velocity: the more reddish is the colour, the greater is this component. Top and bottom boundaries are impermeable to flow. Green line at the bottom is activator (site, covered with TF). Dark gray shows solid clot (fibrin exceeds 3,800 nM). Dark blue shows the rest of the clot, consisting of low concentration fibrin. The detailed kinetics of thrombus formation can be found in Additional files 1 and 2.

Functioning of blood coagulation network was simulated in a two-dimensional region with constant pressure flow as described under Methods. Fig. 1 (see also Additional files 1 and 2) illustrates the design of computational experiments and the general effect of flow on coagulation. The clot initially appeared downstream from the activator and slowly propagated upstream; the clot size downstream from the activator was much greater than the activator size. This clotting downstream from the activator, which may seem not physiological, was most likely caused by lack of thrombomodulin and heparane sulfate on the chamber walls in our simulations. Thrombomodulin present on the endothelium of vessels, is able to inhibit spatial clot propagation [4] and is probably responsible for the lack of uncontrolled fibrin formation downstream from the damage site in vivo. However, this study was focused on the initial stages of clot formation, which are not influenced by thrombomodulin [4]; therefore, we chose not include this additional system into the model. Further, it is interesting to note that the clot is heterogeneous: the high density region (Fn concentration higher than 3,800 nM, i.e. more than 1/2 of fibrinogen concentration) is localized near the activator (dark grey in Fig. 1), while the large thrombus downstream primarily consisted of low density clot (dark blue in Fig. 1).

This following study was concentrated on the most obvious and immediate effect of the flow, the increase of time between TF exposure and the clot appearance. In order to characterize this quantitatively, we introduced the parameter of lagtime, i.e. time required to cover half of the activator with high density fibrin clot (3,800 nM).

**Figure 2:**
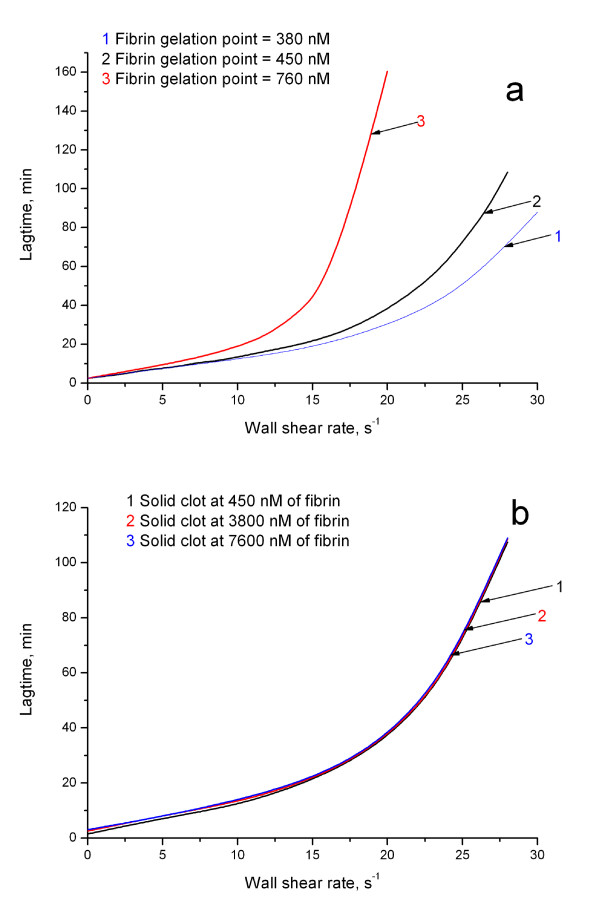
**Dependence of lagtime on flow shear rate at different fibrin gelation parameters**. **(a) **Lagtime dependence is quantitatively, but not qualitatively influenced by the fibrin gelation point. In most simulations, we used fibrin concentration of 450 nM as a point of gelation (black). Here we show that increase of this value up to 760 nM (red) reduces range of flow rates, where coagulation resists flow, but the overall effect of flow does not change. Decreasing the value of fibrin gelation point down to 380 nM (blue) prolongs this range. **(b) **Lagtime is not influenced by the of choice concentration, where we assume fibrin to form a solid clot. Throughout the study, we used value of 3,800 nM. This figure shows the changing this value has no effect.

A series of computational experiments on fibrin thrombus formation at different flow rates was performed. Fig. 2a (black line) shows the above-defined lagtime as a function of wall shear rate. It can be approximated with a function of exponential growth *y *= *a + b e^c·x^, a *= 1.54 ± 0.73, *b *= 2.86 ± 0.32, *c *= 0.129 ± 0.004, where *y *is lagtime and *x *is wall shear rate. From a practical point of view, this means that, at small shear rates (0-13 s^-1^), the lagtime increased slowly with the increase of shear rate from 2.5 min without flow up to 10-20 min. However, this increase became very rapid at higher shear rates, and by 28 s^-1 ^the lag time reached 100-120 min. The results in Fig. 2a can be summarized as follows: 1) clotting is inhibited by flow; 2) this inhibition is strongly non-linear, i.e. the system can resist inhibition caused by flow within a certain range of flow rate, but it is efficiently "turned off" at higher shear rates.

### Dependence of the phenomena on model assumptions

While the vast majority of model parameters are based on experimental data and have been thoroughly validated by comparison with experiments without flow [4], two assumptions were made in this study: specific values for fibrin gelation concentration and the fibrin level to calculate lagtime. In order to estimate the effect of these assumptions, we performed calculations at different values. For this, we introduced two numerical parameters characterizing control of clotting by flow:  (overall inhibition of clotting by flow in the interval of shear rates *i-j*) and *R' *(non-linearity of this inhibition) as defined under Methods.

The increase of the gelation point concentration increased the influence of flow on the system and vice versa quantitatively, but not qualitatively. The principal effect (that coagulation system can resist flow within some range of flow velocities and is suppressed by more rapid flow) remained (Fig. 2a, Table 1). Furthermore, the exact value of fibrin concentration at which clot became solid did not influence the observed flow effect at all (Fig. 2b, Table 1). The most likely explanation is that, when a portion of the activator became protected from the flow by the clot, coagulation reactions within the clot could proceed rapidly, and complete fibrinogen conversion could occur with minimal delays.

**Table 1:** Effect of system perturbation on sensitivity to flow

Condition			*R'*
		***i-j, s***^-1^	
Control, Fn gel concentration 380 nM	0.76	0-28	0.81
Control, Fn gel concentration 760 nM	2.72	0-20	2.05
Control, Fn concentration of solid clot 450 nM	0.97	0-28	1
Control, Fn concentration of solid clot 7600 nM	1.02	0-28	1.01
**Control**	**1**	**0-28**	**1**
Control (activator size 500 μm)	3.35^1^	0-15	2.1^1^
No convection of zymogens	1.03	0-28	1
No convection of enzymes	0	0-28	-
No convection of enzymes except for IIa & Xa	0.39^2^	0-15	0.63^2^
No convection of IIa & Xa	0.01	0-28	-
No Xa-induced VII-TF activation	7.73	0-12	0.33
No IIa-induced activation of V, VII, VIII, XI	1.72	0-12	1.12
Addition of TFPI at 5 nM	5.55	0-12	4.5
Addition of AT-III at 6800 nM	1.38	0-24	1.22
Addition of VIIa at 10 nM	0.07	0-24	0.25

Table 1 indicates how perturbations of the coagulation system alter its behavior (length of coagulation onset) under flow conditions. Two coefficients represent two different types of flow induced influence on this system (see Methods).  shows whether system is suppressed by flow (within range of flow rates from i s^-1 ^up to j s^-1^) better or worth than in normal case ( > 1 or  < 1). Each set of simulation results was approximated, where it was possible, with exponential function of the following type: *y *= *y*_0 _+ *A·e*^*R*·*x*^. Parameter *R' *shows whether system became more sensitive to flow rate variation than in normal case (*R' *> 1 or *R' *< 1).

^1 ^Coefficient calculated relatively to normal case (1000 um activator length)

^2 ^Coefficient calculated relatively to normal case (500 um activator length)

### The effect of flow on coagulation depends on the activator size

**Figure 3:**
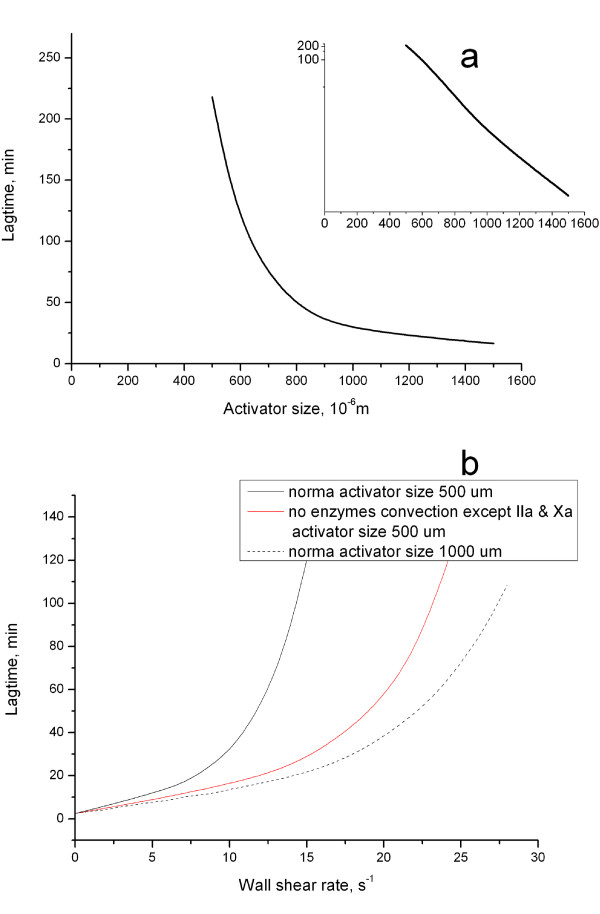
**Dependence of lagtime on activator size**. **(a) **All simulations were carried out as in Fig. 1, except for the difference in activator size. Wall shear rate is 17s^-1^. The inset shows the dependence in a semi-reciprocal scale. **(b) **1) normal case, activator size 500 μm; 2) activator size 500 μm, no convection of all enzymes except for factors IIa and Xa; 3) normal case, activator size 1000 μm.

The simulations described above were carried out with the same activator size of 1000 μm. The damaged area *in vivo *can vary, and it was necessary to find out how the observed phenomena depended on activator size. In order to do this, we determined the dependence of the lag-time on the activator size (Fig. 3a) with wall shear rate being kept constant (17 s^-1^). Increase of the activator size led to the lagtime decrease and vice versa. The dependence was hyperbolical as evidenced by linearity in a semi-reciprocal scale (Fig. 3a inset). There was almost no clotting (at this shear rate) for activators smaller than 500 μm. Additional simulations at different wall shear rates confirmed that all observed phenomena existed at different activation sizes although the specific values of clotting time could change (data not shown). Lagtime dependence on wall shear rate calculated for the 500 μm-long activating patch is presented in Fig. 3b. For this case  = 3.35 and *R' *= 2.1 in comparison with 1000 μm -long activating patch. Influence of flow increased 3-fold and system became 2-fold more sensitive to flow rate variation, although all phenomena remained.

### Chemical reaction as a sensor of flow: a new role for factor VII activation by factor Xa

**Figure 4:**
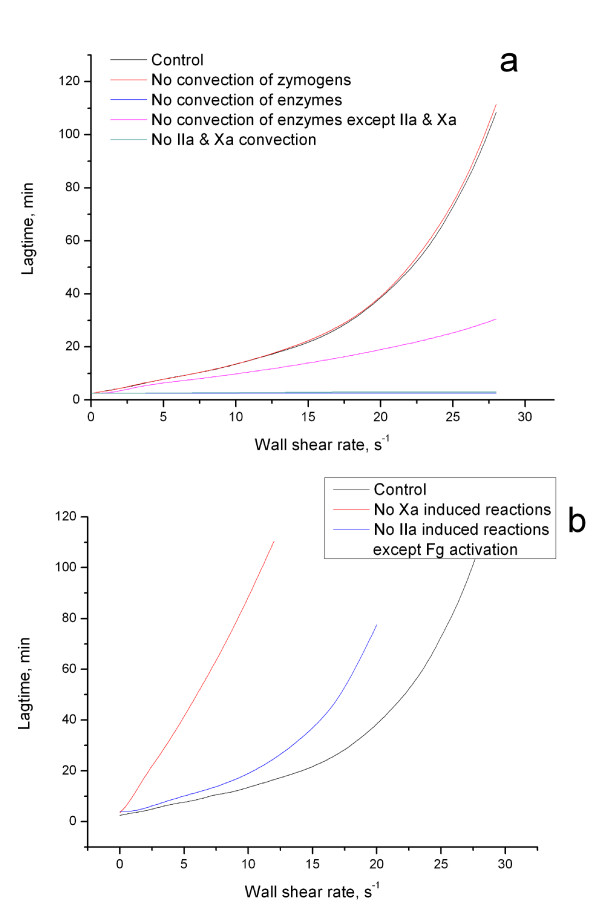
**Dependence of lag time on shear rate is determined by the factor VII activation by factor Xa**. **(a) **Lag time as a function of wall shear rate in the range of shear rates of 0-27 s^-1^. The channel was 1 mm wide, and the activator was 1 mm long. The lines show: 1) normal plasma (black); 2) all zymogens (factors II, V, VII, VIII, IX, X, XI, PC, Fibrinogen) do not move with the flow (red); 3) all enzymes (factors IIa, Va, VIIa, VIIIa, IXa, Xa, XIa, PCa) do not move with the flow (blue); 4) no convection of all enzymes except for factors IIa and Xa (magenta); 5) no convection of factors IIa and Xa only (dark cyan). As can be seen from these curves, the dependence is predominantly determined by the convection of factors IIa and Xa. **(b) **Lag time as a function of wall shear rate without factor IIa-dependent reactions. The lines show: 1) normal plasma (black); 2) no factor IIa-dependent reactions except for fibrinogen cleavage (blue); 3) no factor VII-TF complex activation by factor Xa (red). It can be seen that the factor Xa-dependent activation of the factor VII-TF complex plays a critical role in the formation of the non-linear response of blood coagulation to flow.

In previous sections, we found out that the dependence of clotting initiation on wall shear rate is strongly non-linear. We were also able to show that this phenomenon did not essentially depend on our assumptions and activation conditions. The next step was to figure out the chemical mechanism responsible for this behavior of coagulation system under flow conditions. As a first approach to identify critical elements of the system affected by flow, we carried out virtual experiments, where individual coagulation factors were not carried by flow. In order to "switch off" their convection we removed the respective convection terms from the equations for these factors (see model description). This approach allowed individual coagulation factors to "ignore" the flow thus illustrating the relative contribution of their convection. Red line in Fig. 4a represents the same dependence of lag-time on the wall shear rate in a model experiments without convection of all zymogens (i.e. factors II, V, VII, VIII, IX, X, XI, Fibrinogen). We can see that convection of these factors does not affect the initial phase of clot growth. Coefficients  and *R' *for these cases are presented in Table 1. They show quantitatively that there was no significant difference with normal coagulation.

This was not the case with enzymes (blue line in Fig. 4a). When convection of all enzymes (i.e. factors IIa, Va, VIIa, VIIIa, IXa, Xa, XIa, activated protein C) was "switched off", clot formation within the total range of flow rates was exactly the same as for the zero shear rate. Neither lagtime (Fig. 4a) nor clot shape (data not shown) depended on wall shear rate.  = 0 and *R' *could not be calculated at all, as the lagtime dependence on wall shear rate could be approximated with a line *y *= 2.5. The system became completely insensitive to flow. Thus, it is the removal of active factors by flow that changes the coagulation system behavior under flow conditions.

Further analysis carried out by analogy revealed specific enzymes responsible for the effect. "Switching off" convection of factors IIa and Xa only had the same effect as that of all enzymes taken together (magenta line in Fig. 4a, table 1). *R' *could not be calculated, and  was very low (0.01). Additional analysis showed that individual contributions of factors IIa and Xa to the phenomenon were comparable (data not shown). When convection of all enzymes except for factors IIa and Xa (i.e. Va, VIIa, VIIIa, IXa, XIa, activated protein C) was turned off (dark cyan line in Fig. 4a, Table 1), the influence of flow on coagulation system was reduced approximately 2.6-fold ( = 0.39), while sensitivity to flow rate variation was reduced by a factor of 1.6 (*R' *= 0.63). This remaining difference was due to factor Va convection: turning on factor Va convection in this system made the difference with normal case minimal (data not shown). Summarizing, these simulations provide evidence that it is convection of factors IIa and Xa that determines the effect of system sensitivity to flow rate variation.

After identification of the specific factors, removal of which by flow is most important for the regulation of coagulation system behavior under flow conditions, we analyzed the reactions, wherein these factors participate by their removal from the system. Thrombin-mediated cleavage of fibrinogen and prothrombin activation by factor Xa are essential and critical components of the coagulation system, and their removal from the reaction scheme could not be attempted. Other important reactions involving these enzymes are positive feedback loops of factors V, VII, VIII, XI activation by thrombin, and of the factor VII-TF complex activation by factor Xa [12]. We found out that "switching off" all positive feedbacks catalyzed by thrombin caused moderate increase of  and almost did not change *R' *(blue line in Fig. 4b). Thus, although system was slightly better suppressed by flow, its sensitivity to flow rate variation remained on the same level (*R' *= 1.12). This was not the case for factor Xa-catalysed reactions: "turning off" the factor VII-TF complex activation (red line in Fig. 4b) made the system 3-fold less sensitive to flow rate variation (*R' *= 0.33), and it was suppressed by flow 7.7-fold better.

We propose the following explanation of observed phenomena: the onset of blood coagulation under flow conditions is governed by the positive feedback of extrinsic tenase activation by factor Xa. When flow does not influence this feedback (as it was in the case of no convection of factors Xa and IIa), the duration of clotting initiation phase becomes independent of flow rate. When the feedback does not function (factor Xa does not activate complex VII-TF to its active form, extrinsic tenase) flow suppresses coagulation, although this influence varies little as flow rate is increased. But in normal case, flow gradually switches off feedback extrinsic tenase activation. This not only causes lagtime increase, but also makes this increase non-linear.

### Flow-dependent regulation of extrinsic tenase production

**Figure 5:**
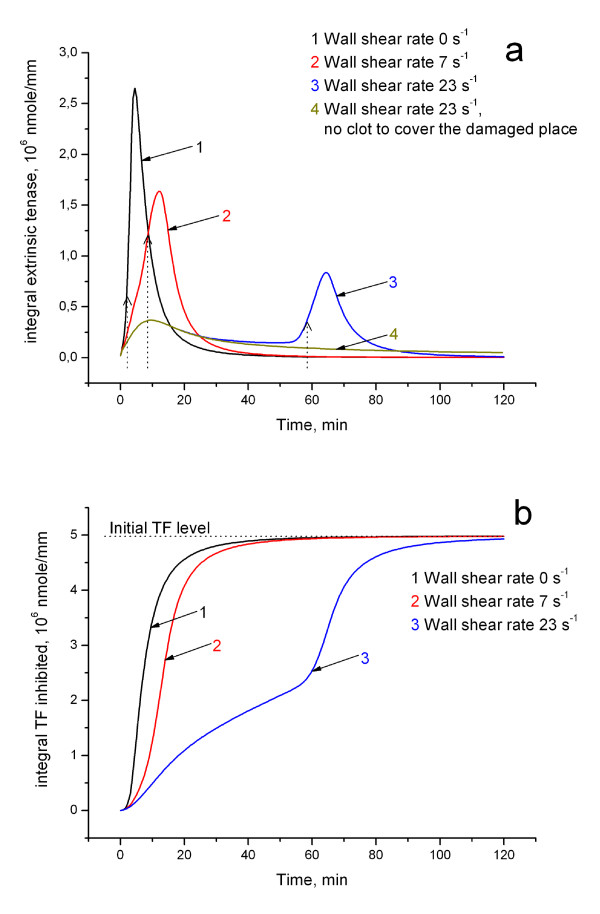
**Kinetics of integral extrinsic tenase and inhibition of TF**. **(a) **Integral extrinsic tenase kinetics under different flow conditions (stagnant plasma (black), wall shear rate 7s^-1 ^(red) and 23s^-1^(blue)) and in the case of no clot formation (dark yellow). Dotted arrows show the lagtime. **(b) **Integral kinetics of TF inhibition under different flow conditions. Dotted line shows initial TF level.

Extremely long lagtimes observed in the simulations in the presence of flow can appear unusual, because TFPI usually limits the lifetime of extrinsic tenase to several minutes [13]. To better investigate the events occurring during the initiation stage of clotting in flow, the integral extrinsic tenase kinetics was simulated and compared for three different cases: a) stagnant plasma (wall shear rate 0 s^-1^); b) moderate flow (wall shear rate 7 s^-1^); c) rapid flow (wall shear rate 23 s^-1^). The results are shown in the Fig. 5a. Lagtimes are marked with arrows. In stagnant plasma, almost all extrinsic tenase is inhibited by the 24^th ^minute of experiment (integral value of extrinsic tenase is lower than 0.1 × 10^-6 ^nmole/mm). The moderate flow decreased the rate of extrinsic tenase accumulation and the maximal concentration achieved, but it also prolonged its existence up to 31 minutes. This was even better seen in the extrinsic tenase kinetics under the rapid flow conditions. During the first 10 minutes, TF could bind factor VIIa circulating in blood at a small concentration. However, formation of the peak was inhibited by flow because of the removal of factor Xa and the resulting decrease of TF-VII activation. Subsequently, slow inhibition of extrinsic tenase began, but then a new peak appeared, and it causes the onset of coagulation. This peak appears when about a half of the activator is covered by the clot (time, when initiation phase considered to be finished) and thus protected from the flow. If thrombin-induced fibrinogen conversion was removed from the model, and thus no clot could be formed, the second peak disappeared.

Fig. 5b represents the kinetics of TF inhibition at different shear rates. It illustrates that flow actually prolongs the lifetime of extrinsic tenase. Summarizing, the calculations in Fig. 5 show that flow plays a dual role in the regulation of extrinsic tenase. On the one hand, extrinsic tenase formation is slowed down, and clot is slower to form. On the other hand, this protects TF from inhibition (it can be inhibited only in complex with VIIa) and thus flow can prolong the life time of extrinsic tenase and allow clot initiation later.

### Possible tests of the model predictions

Although the "flow-dependent trigger" obtained in our model was in agreement with experimental evidence [10,14,15] and did not require any hypotheses about unknown reactions, its mechanism remains to be experimentally confirmed. The most direct way to do this would be to study coagulation in a flow chamber using mutant molecules of factors X and VII that would be unable to participate in some of the feedback reactions. However, there are simpler experiments that can be undertaken to test tis mechanism, and we attempted to predict them with the help of the model.

**Figure 6:**
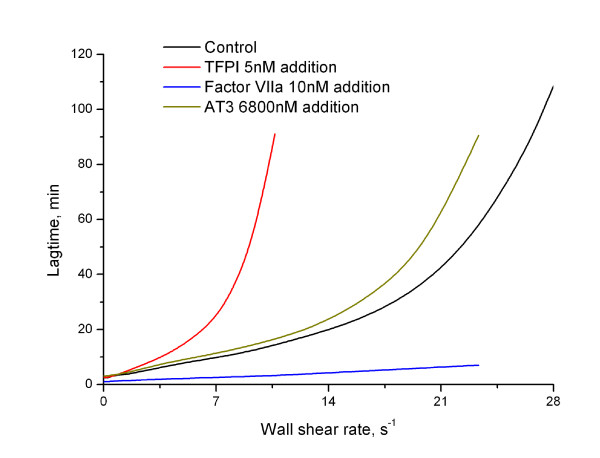
**The dependence of lag time on shear rate is determined by the factor VII activation, TFPI- and AT3-mediated inhibition**. Lag time is shown as a function of shear rate for normal plasma, and for plasma supplemented with factor VIIa, TFPI or AT3 as indicated in the legend. Addition of factor VIIa results in dramatic lag time decrease and the dependence becomes linear (blue). Addition of TFPI shifts the threshold to the region of lower shear rates (red). Addition of AT3 slightly increased lag time (dark yellow), compare to normal case. These results indicate that factor VII activation and extrinsic tenase inhibition with TFPI are indeed the controlling mechanisms determining behavior of coagulation in the presence of flow.

Addition of 10 nM factor VIIa (equal to the plasma concentration of factor VII) in the model dramatically shortened the lagtime (its suppression by flow was reduced 14-fold,  = 0.07), and decreased sensitivity to flow rate variation 4-fold (*R' *= 0.25) (Fig. 6). That the sensitivity to flow rate variation in plasma, where factor VII is already significantly activated, should be very low, would be the most important and clear confirmation of the principal conclusion of this study: that it is the positive feedback of factor VII activation that regulates the dependence of clot lag time on wall shear rate. In this experiment, initial ratio of active factor VIIa to inactive factor VII would be 1:1, so 1/2 of all complexes of factor VII/VIIa with TF would be extrinsic tenase (instead of 1% in normal plasma). This can be interpreted as increase of extrinsic tenase activation feedback speed, which allows to sufficiently neglect influence of flow on the system.

On the other hand, addition of 5 nM TFPI (3-fold increase of the normal plasma concentration) should result in a dramatic increase of suppression by flow ( = 5.55) (Fig. 6), and the system sensitivity to flow rate variation should also increase (*R' *= 4.5). TFPI would have very little effect in the absence of flow, similar to the lack of effect of factor VII activation without flow. Increase of TFPI concentration would lead to better inhibition of the feedback, thus system would be more sensitive either to flow rate variation and suppression by flow. We propose that TFPI concentration is a determining factor for the range of flow rate where the coagulation system can form a clot.

As a possible control, addition of 6800 nM antithrombin III (3-fold increase of the normal plasma concentration) should result in a very moderate increase of suppression by flow ( = 1.38), definitely much less than in the case of TFPI addition (Fig. 6), and should not change the sensitivity to flow rate variation sufficiently (*R' *= 1.22). So we propose that antithrombin III-induced regulation of the early stages of coagulation in flow is moderate.

## Discussion

**Figure 7:**
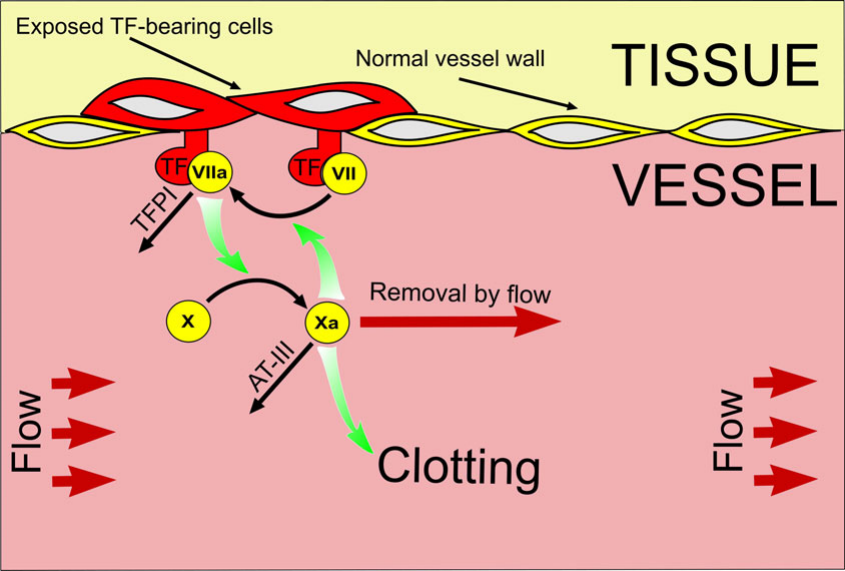
**Regulation of fibrin clotting by flow via the factor VII activation by factor Xa**. The diagram illustrates the mechanisms controlling initiation of coagulation in the presence of flow. Factor VIIa initially present in plasma binds to TF and activates factor X; it is also inhibits by TFPI. Factor Xa can activate TF-bound factor VII in a positive feedback manner. In the absence of flow, both inhibition by TFPI and activation by factor Xa are not significant, because high concentrations of TF present on fibroblast rapidly bind factor VIIa; they do not need additional factor VII activation and are not particularly sensitive to factor VIIa-TF complex inhibition. When blood flow is present, factor Xa is rapidly removed, and the rate of factor X production becomes insufficient to create fibrin clot (factor VIIa-TF complex being inhibited by TFPI). This is when factor Xa-dependent feedback becomes important: factor Xa activates factor VII and increases its own production, counteracting the effects of flow. At higher shear rates, this feedback is insufficient, and factor Xa production is strongly inhibited. Thus, inhibition of factor VIIa-TF complex by TFPI and factor VII activation by factor Xa combine to create a threshold-like response of the system in flow: rapid clotting at low shear rates and almost no thrombus formation at higher shear rates.

The principal conclusions of this study can be depicted as a concept of clotting regulation by flow shown in Fig. 7. According to this concept, the critical step of blood coagulation control in the presence of flow is drag-out of factor Xa from the site of vascular damage by flow. Without flow, quantities of extrinsic tenase formed by TF and trace amounts of factor VIIa already present in normal plasma are sufficient to generate enough factor Xa in order to initiate clotting reaction. Additional activation of factor VII by factor Xa is not needed, and the role of this reaction in the absence of flow is small. Further, inhibition of extrinsic tenase by TFPI in the absence of flow is not essential either, because of the same reasons.

In the presence of flow with low shear rates, more extrinsic tenase is needed to clot plasma because factor Xa is removed by flow. However, additional factor VIIa-TF complex produced by factor Xa from factor VII-TF complex can meet these requirements. Because of this positive feedback, blood coagulation is almost not sensitive to flow at low wall shear rates: with shear rate increase, lagtime increases very slowly. The region of this low sensitivity is determined by the balance of positive feedback of factor VII activation and TFPI-mediated inhibition: the lagtime is sensitive to these reactions in the presence of flow (Fig. 4). This result disagrees with the theoretical prediction of [16], where TFPI was found not to influence coagulation in flow at all. We suppose that this disagreement was caused by different ways of modeling TFPI inhibition [2].

Finally, at higher shear rates this mechanism is no more able to support clotting: factor Xa is removed very rapidly and is not able to activate factor VII-TF complex. Lag time becomes very long and is rapidly increasing as an exponential function of wall shear rate.

In other words, this concept can be summarized as follows: positive feedback activation of factor VII-TF complex by factor Xa, inhibition of factor VIIa-TF complex by TFPI and factor Xa removal by flow from the activation region together constitute a special controlling mechanism, which makes blood coagulation insensitive to flow at low shear rates, and almost completely switches clotting off at higher shear rates.

This concept agrees well with the theoretical prediction of Beltrami and Jesty that a biochemical system composed of two enzymes reciprocally activating each other in the presence of flow, one enzyme being membrane-bound and another being soluble, will depend on the wall shear rate in a threshold manner[17]. A marked difference between that prediction and the result obtained here for coagulation system is that our, more complex system does not actually have a clear threshold: it is just a steep exponential dependence, which can, however, serve the same regulatory purposes.

Generally speaking, flow can exert its effects by either drag-in or drag-out of the components of coagulation. Previous studies somewhat disagree in the relative contribution of these phenomena. Experiments performed using a reconstituted system and a continuous flow reactor have shown that drag-in of rapidly consumable coagulation factor predecessors (such as factor VIII) can be very important and enhance coagulation [18]. Computer simulations of fibrin polymerization [19] and enzyme catalysis on the channel wall [17] in flow suggested that it is the drag-out of these components which is the most important mechanism of flow action. Our calculations performed in a detailed model of coagulation system agree with the latter conclusion: removal of coagulation factors by flow is the predominant mechanism. Moreover, we were able to identify two most important factors that are removed, factor Xa and thrombin. Convection of other factors contributes negligibly.

Previous studies do not completely agree in their conclusions on flow effect in coagulation. Although there are reports that blood flow can have some beneficial effect on blood coagulation, at least in purified systems (for example, the rate of factor X activation by extrinsic tenase under high shear rate conditions is increased [18,20]), it is more or less generally accepted that the overall effect of flow on clotting is inhibitory as can be seen from both flow chamber perfusion experiments and in vivo studies [10,11,15,21,22]. The mechanisms of this effect are not clear either. A recent mathematical modeling study has shown that flow can inhibit fibrin polymerization [19] and that this inhibition can give results similar to those obtained experimentally [15]. The same group showed that flow-mediated removal of factors IXa and Xa inhibits the initiation of coagulation [16].

We studied the behavior of plasma coagulation depending on the wall shear rate in the range of 0-28 s^-1^, and found out that at rates higher than 17-28 s^-1 ^plasma coagulation is almost inhibited by flow. It should be considered that this range of wall shear rates significantly depends on the activator size and the chosen mechanism of gelation. If we take into consideration that most researchers estimate physiological range of wall shear rates as 25-6500 s^-1 ^according to [23] then we cannot escape the conclusion that the clotting system function is actually designed to minimize fibrin thrombi formation under physiological conditions. The problem of fibrin polymerization in flow requires further exploration, because it can essentially affect the range of shear rates where clotting is possible; the qualitative phenomenon, however, should not change because it is determined by flow effect on factors IIa and Xa.

Summarizing, our results suggest that roles of the initial reactions of the extrinsic pathway might be somewhat reconsidered. In particular, this is relevant for factor VII-TF complex activation by factor Xa and factor VIIa-TF complex inhibition by TFPI. In the absence of flow, these reactions do not have clear roles. Although they can influence clotting activated with diluted TF in vitro [13,24], their contribution in the presence of TF highly concentrated on fibroblasts is negligible in the conditions of this study: formation of extrinsic tenase is sufficient without any need of additional factor VIIa, and inhibition by TFPI cannot significantly delay clotting [13]. On the other hand, in the presence of flow, which removes factor Xa (both the product and the activator of extrinsic tenase) these reactions become critically important and completely shape the response of the system.

## Conclusions

Removal of factor Xa by flow is the predominant mechanism of flow influence on the initial stage of blood coagulation. Together with TFPI-induced inhibition of extrinsic tenase complex, these mechanisms provide strongly non-linear response making coagulation insensitive to flow at low shear rates and almost preventing coagulation at higher rates. Although elucidation of these purposes is beyond the scope of this study, it might be speculated that this mechanism can protect the vascular system from formation of fibrin thrombi at high shear rates. However, when blood flow is reduced (e.g., as a result of platelet plug formation), the clotting in the area without flow can proceed rapidly.

## Methods

### Mathematical model

A detailed mathematical model of blood coagulation cascade previously developed and verified by our group [4] for clotting of plasma without flow was used with modifications that included addition of a second dimension, convection terms, Navier-Stokes equations, and the process of fibrin polymerization as described below. The model consisted of 28 partial differential equations describing biochemical reactions, diffusion and convection of the reactants. All initial concentrations and kinetic constants used in the model were obtained from experimental reports, and no adjustment was performed.

The model simulated coagulation in a two-dimensional rectangle region (1,000 × 6,000 μm) with laminar flow. Activation patch (1,000 μm) covered with tissue factor (TF) was located on one of the walls of the simulated channel. When coagulation was activated, it led to fibrin network formation. We assumed the rate of fibrin polymerization to be sufficiently high so that each fibrin molecule was rapidly incorporated into the network; thus, we neglected the effect of flow on fibrin fibers formation. The permeability of this network was assumed to depend on fibrin concentration. While fibrin concentration was below the point of gelation, the permeability was 1, and it reduced to 0 when the fibrin concentration exceeded the critical concentration. Although impermeable to flow, fibrin gel in the model did not hinder diffusion [25] and was assumed to have no effect on the reactions of coagulation except for thrombin adsorption onto fibrin. The detailed description of the model is available in Additional file 3 and program realization of mathematical simulation based on this model is presented in Additional file 4.

### Modular analysis

In order to identify reactions responsible for the regulation of clotting by flow, we introduced a parameter characterizing the time of initiation phase. Lagtime was defined as the time when clot reached the middle of the activator. We measured the dependence of lagtime on flow rate and obtained the normal behavior of the system, so that we could trace its variations when perturbing the system. In order to quantify lagtime dependence on flow rate we used the following coefficient:(1)

This is comparative flow influence coefficient in range of flow rates *i*-*j *(from *i *s^-1 ^up to *j *s^-1^). Each set of simulation results was approximated with the exponential function *y *= *y*_0 _+ *A·e*^*R*·*x*^. Parameter(2)

was used as relative flow rate variation sensitivity coefficient.

The first coefficient indicates the integral flow induced lagtime growth within the certain range of flow rates, and the second coefficient indicates the increase of the rate of lagtime growing in the dependence lagtime versus flow rate. Based on values of these parameters, we could decide whether a certain part of coagulation system was sufficient for forming its behavior under flow conditions.

## Authors' contributions

AMS developed the mathematical model, created the program to solve the model, designed the study, performed research, analyzed the data and wrote the paper. ESL developed the mathematical model and created the program to solve the model. MAP designed the study, analyzed the data and wrote the paper. FIA conceived the study, designed the study, analyzed the data and wrote the paper.

All authors read and approved the final version of the manuscript.

## Supplementary Material

Additional file 1 **Thrombus formation without flow**. Movie representing simulation of thrombus in stagnant plasma (movie time/real time is 1/90). Click here for file

Additional file 2 **Thrombus formation under flow conditions**. Movie representing simulation of thrombus in flowing plasma (movie time/real time is 1/90). Wall shear rate is 17 s^-1^. Click here for file

Additional file 3 **Mathematical model of blood coagulation in the presence of flow**. Description of the mathematical model and of the numerical methods. Click here for file

Additional file 4 **Program code files**. Source files of program realization of blood coagulation modeling under flow conditions. Click here for file
