# Supplementary material for: Blood flow controls coagulation onset via the positive feedback of factor VII activation by factor Xa
Source: BMC Syst Biol. 2010 Jan 26;4:5. doi: 10.1186/1752-0509-4-5 (PMC2823678; doi:10.1186/1752-0509-4-5)
Supplement: Additional file 3 — Mathematical model of blood coagulation in the presence of flow. Description of the mathematical model and of the numerical methods. [file 1752-0509-4-5-S3.DOC]

Additional file 3

***Mathematical model of blood coagulation in the presence of flow***

### Contents

[Contents 1](#__RefHeading___Toc247265105)

[Model Description 1](#__RefHeading___Toc247265106)

[Hydrodynamic Equations 2](#__RefHeading___Toc247265107)

[Biochemical Equations 3](#__RefHeading___Toc247265108)

[Model Parameters 10](#__RefHeading___Toc247265109)

[Numeric methods 15](#__RefHeading___Toc247265110)

[Solving Navier-Stokes Equations 15](#__RefHeading___Toc247265111)

[Solving Biochemical Equations 19](#__RefHeading___Toc247265112)

[Estimation of Calculation Errors 21](#__RefHeading___Toc247265113)

[Reference List 23](#__RefHeading___Toc247265114)

## Model Description

*Model assumptions.* Mathematical model of coagulation in flowing blood plasma consists of 28 partial reaction-convection-diffusion differential equations. It is based on the mathematical model of clotting in a one-dimensional reaction-diffusion system previously developed by our group [1]. All assumptions of that study hold here as well. The original model is modified to describe coagulation in a two-dimensional region in the presence of flow by addition of: a) a second dimension, b) Navier-Stokes equations, and c) clot formation that changes flow boundary.

Plasma is assumed to be viscous and incompressible. We also assume that, within the range of shear rates considered, fibrin polymerization occurs sufficiently rapidly so that fibrin neither diffuses nor is transported by flow. Until the concentration of fibrin is below the point of gelation (n_gp_) the fibrin clot is permeable to flow; as soon as it exceeds n_gp_, the clot becomes impermeable to flow. All model parameters, unless otherwise stated, are the same as in [1].

*Simulation region.* Thrombus formation is simulated in a two-dimensional L_x_×L_y_ rectangular area, which physically corresponds to a channel L_x_ long and L_y_ wide between two planes that are infinitely long in the *z* direction. Channel walls are impermeable for plasma. On the bottom wall (L_y_=0) of the area, there is an activation patch of the length L_a_ covered with tissue factor at a surface density of $\sigma_{TF}$.

*Fibrin clot formation.* For our calculations, we hold the following assumptions on clot formation and its interaction with the flow. We assume fibrin polymerization to occur sufficiently rapidly so that fibrin neither diffuses nor is transported by flow. Also, fibrin clot permeability depends on fibrin concentration. While the concentration of fibrin is below the point of gelation (n_gp_) the fibrin clot is permeable to flow; as soon as it exceeds n_gp_, the clot becomes impermeable to flow.

*Interaction of clot and flow.* It is necessary to couple field of flow velocities and reagents concentration distribution. Flow velocity depends on fibrin concentration due to formation of impermeable clot; reagent concentration changed due to flow transfer. We use quasistationary approach to couple changes in flow velocity and reagents concentration. We take into consideration that the rate of stationary flow stabilization is much higher than clot growth rate.

Let us estimate validity of the quasistationary assumption.

Growing clot causes tangential discontinuity in the field of velocity of incompressible viscous liquid. Smoothing of the perturbation extends to the distance $\Delta l\sim\sqrt{\nu\cdot t}$ at the time *t*[2]. Here, *ν* is the kinematic viscosity. For blood plasma, $\nu\approx2\cdot10^{-6}$m^2^/s. Clot growth rate is 0.67∙10^-6^ m/s [3]. Let t=1 s. Then, during this time, a clot can increase its size by a value of $\Delta h\sim10^{-6}$m. The respective area of smoothing $\Delta l\sim10^{-3}$m is 3 orders of magnitude larger than the area of clot propagation. In other words, the rate of flow stabilization is 3 orders of magnitude higher than the rate of clot growth. Because of this, we can solve Navier-Stokes equations independently from the equations describing reagent concentration changes.

### Hydrodynamic Equations

Navier-Stokes equation is used to describe incompressible viscous liquid motion:

S1

where $\vec{v}$ is the velocity of flow, *p* is the pressure, $\nu$ is the kinematic viscosity of liquid, $\rho$ is the fluid density. Combined with the continuity equation, which in the case of incompressible flow can be written as follows:

, S2

these equations have only one solution for each set of initial and boundary conditions.

*Initial and boundary conditions* are:

$$\begin{matrix} \left.v_{x}\left(y\right)\right|_{t=0}=\frac{g}{L_{y}}y\left(L_{y}-y\right), \\ \left.v_{y}\right|_{t=0}=0, \\ \left.\frac{\partial P}{\partial x}\right|_{t=0}=-\frac{2g\xi}{L_{y}}; \\ \left.v\right|_{y=0,y=L_{y}}=0, \\ \left.\frac{\partial v_{x}}{\partial x}\right|_{x=0,x=L_{x}}=0, \\ \left.v_{y}\right|_{x=0,x=L_{x}}=0 \\ \left.P\right|_{x=0}=\frac{2g\xi L_{x}}{L_{y}}, \\ \left.P\right|_{x=L_{x}}=0; \end{matrix}$$

S3

S4

S5

S6

S7

S8

S9

S10

Notation: $v$ is flow velocity; $v_{x},v_{y}$ are horizontal and vertical components of flow velocity, respectively; $L_{x},L_{y}$ are spatial dimensions of the simulation region; *g* is wall shear rate; $\xi$ is the plasma viscosity; *P* is pressure. In all calculations in the paper, the geometric parameters are: $L_{x}=6 \text{mm}$, $L_{y}=1 \text{mm}$, $\xi=2\frac{\text{kg}}{\text{mm}\cdot\text{s}}$, $g=0.30 \text{s}^{-1}$.

Eq. S2-S4 are initial conditions. They define initial parabolic profile of $v_{x}$(S1), zero value of $v_{y}$(S2), and linear gradient of pressure *P* (S3). Use of these initial conditions makes solution of the Navier-Stokes equations more rapid, as they are close to the solution for the case of laminar flowing liquid between two parallel planes.

Eq. S5-S9 are boundary conditions. Eq. S5 defines condition of no slip on the walls; eq. S6 defines free flow on both ends of simulation area; eq. S7 defines undisturbed flow in y direction on both ends of the simulation area; eq. S8-S9 define condition of constant pressure expressed in the used notations.

Numeric methods used to solve Navier-Stokes equations are described in the second part of this supplementary file, named *Numeric methods*.

### Biochemical Equations

Continuity equation describing changes in concentration *φ* caused by diffusion, flow transfer and production in chemical reactions can be presented as follows:

S11

where $\vec{j}$ is function describing flux of *φ*, and *f* is function describing production of *φ*.

, S12

where $\vec{j}_{Diffusion}$ is diffusive flux of *φ*; $\vec{j}_{Convection}$ is convectional flux of *φ*.

According to the Fick's first law,

, S13

where *D* is diffusion coefficient, and

, S14

where $\vec{v}$ is flow velocity.

Thus,

S15

For a stationary flow of incompressible liquid

,

so

, S16

and this is an equation of reaction-diffusion-convection type.

As plasma coagulation is a spatially heterogeneous process, some reactions take place in the whole volume, while others occur only on the activation surface. We assume that there is neither factor diffusion nor convection within this surface.

*Notation.* Variable [*F*] denotes factor F concentration. [*F^F^*] denotes concentration of free factor F in the solution. [*F^B^*] denotes concentration of factor F bound to the activated platelets. The surface concentration of factors on the activation surface is denoted σ. Notations for rate constants of factors F_1_ and F_2_ association and dissociation are $k_{a}^{F_{1}, F_{2}}$ and $k_{d}^{F_{1}, F_{2}}$. $K_{d}^{F_{1}, F_{2}}$ is the equilibrium dissociation constant. $k_{cat}^{F_{1}, F_{2}}$, $K_{M}^{F_{1}, F_{2}}$, and $k_{eff}^{F_{1}, F_{2}}$ are respectively catalytic, Michaelis and effective rate constants for factor F_1_ catalysis by factor F_2_. Upper index "local" denotes a local, two-dimensional constant for reactions on phospholipide membranes. *D_F_* is the diffusion coefficient of factor F.

*Differential equations:*

$\frac{d\sigma_{VIIa-TF}}{dt}$= $\left(k_{a}^{VIIa, TF}\cdot\left.\left[VIIa\right]\right|_{y=0}\cdot\sigma_{TF}-k_{d}^{VIIa-TF}\cdot\sigma_{VIIa-TF}^{F}\right)$ + $\frac{k_{cat}^{VII-TF, IIa}\cdot\sigma_{VII-TF}\cdot\left.\left[IIa^{F}\right]\right|_{y=0}}{K_{M}^{VII-TF, IIa}+\left.\left[IIa^{F}\right]\right|_{y=0}}$+ +$k_{eff}^{VII-TF, Xa}\cdot\sigma_{VII-TF}\cdot\left.\left[Xa^{F}\right]\right|_{y=0}$ – $k_{a}^{VIIa-TF, Xa-TFPI}\cdot\left.\left[Xa-TFPI\right]\right|_{y=0}\cdot\sigma_{VIIa-TF}^{F}$ –

S17

–

= – –

S18

–

= – –

S19

–

= –

S20

– +

= – –

S21

–

=+

S22

+ + +

+ –

= – –

S23

– – –

=+ + + +

S24

+ + –

– –

– –

–

= – –

S25

– –

– –

=+ + + –

S26

–

= – –

S27

–

=

S28

S29

= –

S30

= + –

= –

S31

= + –

S32

– –

= –

S33

= + +

S34

+ –

–

= – – –

S35

= +

+ –

S36

–

= –

S37

– –

–

= –

S38

– – –

– – –

–

=+ –

S39

–

= –

S40

*Algebraic equations:*

$\sigma_{VIIa-TF}^{F}$=$\frac{\sigma_{VIIa-TF}}{1+\frac{\left.\left[IX\right]\right|_{y=0}}{K_{M}^{IX, VIIa-TF}}+\frac{\left.\left[X\right]\right|_{y=0}}{K_{M}^{X, VIIa-TF}}}$

S41

=

S42

=

S43

=

S44

=

S45

[*Xa–Va^B^*]= $\frac{\left[Xa\right]\cdot\left[Va^{B}\right]}{K_{d}^{Xa-Va}\cdot\left(1+\frac{\left[PS\right]}{K_{i}^{Va, PS}}+\frac{\left[Xa\right]}{K_{d}^{Xa-Va}}\right)+\left[Va^{B}\right]}$

S46

S47

[*Xa^F^*]=[*Xa*] – [*Xa–Va^B^*]

$\left[X^{B}\right]$=$\frac{\left[X\right]\cdot\left(N_{l}+N_{m}\right)\cdot n^{X\left(II\right)}}{K_{d}^{X}\cdot\left(1+\frac{\left[X\right]}{K_{d}^{X}}+\frac{\left[II\right]}{K_{d}^{II}}\right)}$

S48

[*IIa^F^*]=$\frac{\left[IIa\right]}{1+\frac{\left[Fg\right]+\left[Fn\right]}{K_{M}^{Fg, IIa}}}$

S49

S50

[*II^B^*]=$\frac{\left[II\right]\cdot\left(N_{l}+N_{m}\right)\cdot n^{X\left(II\right)}}{K_{d}^{II}\cdot\left(1+\frac{\left[X\right]}{K_{d}^{X}}+\frac{\left[II\right]}{K_{d}^{II}}\right)}$

$$\frac{\left[PS\right]\cdot\left[Va^{B}\right]}{K_{i}^{Va, PS}\cdot\left(1+\frac{\left[PS\right]}{K_{i}^{Va, PS}}+\frac{\left[Xa\right]}{K_{d}^{Xa-Va}}\right)}$$

S51

S52

[*Va^B^*]=$\frac{\left[Va\right]\cdot\left(N_{l}+N_{m}\right)\cdot n^{Va}}{K_{d}^{Va}+\left[Va\right]}$

S53

$\frac{\left[Va\right]\cdot\left(N_{l}+N_{m}\right)\cdot n^{Va}}{K_{d}^{Va}+\left[Va\right]}$ – – [*Xa–Va^B^*]

S54

[*XIa^B^*]=$\frac{\left[XIa\right]\cdot\left(N_{l}+N_{m}\right)\cdot n^{XIa}}{K_{d}^{XIa}+\left[XIa\right]}$

S55

[*XIa^F^*]= [*XIa*] – [*XIa^B^*]

*Initial conditions:*

$$\left.[F]\right|_{t=0}=[F_{0}]$$

S56

*Boundary conditions:*

$$\left.[F]\right|_{x=0}=\left.[F]\right|_{t=0}$$

S57

S58

$\left.\frac{\partial[F]}{\partial y}\right|_{y=0,y=L_{y}}=0$, except for reactions on the activation patch

S59

Only for factors IXa, IX, Xa, X, Xa-TFPI, TFPI the presence of activation on the activation patch caused changes in boundary conditions:

$$D_{VIIa}\cdot\left.\frac{\partial\left[VIIa\right]}{\partial y}\right|_{y=0,x\in A.P.}=\left(k_{a}^{VIIa, TF}\cdot\left.\left[VIIa\right]\right|_{y=0,x\in A.P.}\cdot\sigma_{TF}-k_{d}^{VIIa-TF}\cdot\sigma_{VIIa-TF}^{F}\right)$$

S60

S61

S62

S63

S64

S65

S66

S67

These reactions take place only near activation patch (*A.P.*). We neglect diffusion in the *x* dimension, as boundary lies on membrane and we assume that all reagents came there directly from the adjacent areas.

### Model Parameters

We used the following set of model parameters, as it was shown in the previous work of our group [1] that the one-dimension reaction-diffusion model of blood coagulation was in good correspondence with experimental data, while using this model parameters set.

Table S1. Model parameters: initial conditions

| 1. Initial concentrations of model variables | | | | | |
| --- | --- | --- | --- | --- | --- |
| Factor | Surface density,  10^6^ nmoles/mm^2^ | Factor | Concentration, nM | Factor | Concentration, nM |
| $\sigma_{VIIa-TF}$ | 0 | VIIa | 0.1 | Va | 0 |
| $\sigma_{VII-TF}$ | 0 | VII | 10 | V | 20 |
| $\sigma_{TF}$ | 5×10^-2^ | IXa | 0 | APC | 0 |
|  |  | IX | 90 | PC | 60 |
|  |  | Xa | 0 | Xa–TFPI | 0 |
|  |  | X | 170 | TFPI | 2.5 |
|  |  | IIa | 0 | AT-III | 3400 |
|  |  | II | 1400 | XIa | 0 |
|  |  | Fn | 0 | XI | 30 |
|  |  | Fg | 7600 |  |  |
|  |  | VIIIa | 0 |  |  |
|  |  | VIII | 0.7 |  |  |
| 2. Constant concentrations of the model | | | | | |
| Factor | Concentration, nM | Factor | Concentration, nM | Factor | Concentration, nM |
| N_m_ | 4.5·10^-5^ | α_1_AT | 40000 | PCI | 88 |
| N*_l_* | 3·10^-5^ | α_2_AP | 1100 | C1I | 1700 |
| α_2_M | 3000 | HC-II | 1400 | PS | 346 |
| n_gp_ | 450 |  |  |  |  |

Table S2. Model parameters: kinetic constants

| Constant | Value | Reference |
| --- | --- | --- |
| 1. initiation | | |
| $k_{a}^{VIIa, TF}$, $k_{d}^{VIIa-TF}$ | 2.75 nM^-1^min^-1^, 1.1 min^-1^ | [4,5]* |
| $k_{a}^{VII, TF}$, $k_{d}^{VII-TF}$ | 2.75 nM^-1^min^-1^, 1.1 min^-1^ | [4,5]* |
| $k_{cat}^{VII-TF, IIa}$, $K_{M}^{VII-TF, IIa}$ | 3.66 min^-1^, 2700 nM | [6] |
| $k_{eff}^{VII-TF, Xa}$ | 0.4 nM^-1^min^-1^ | [7] |
| $k_{cat}^{VII, IIa}$, $K_{M}^{VII, IIa}$ | 3.66 min^-1^, 2700 nM | [6] |
| 2. Cascade backbone | | |
| $k_{cat}^{IX, VIIa-TF}$, $K_{M}^{IX, VIIa-TF}$ | 6.8 min^-1^, 250 nM | [8] |
| $k_{d}^{Xa-VIIa-TF}$ | 770 min^-1^ | [9] |
| $k_{eff}^{IX, VIIa}$ | 0.0002 nM^-2^min^-1^ | [10]* |
| $k_{cat}^{IX, XIa}$, $K_{M}^{IX, XIa}$ | 5.8 min^-1^, 200 nM | [11] |
| $k_{cat}^{X, VIIa-TF}$, $K_{M}^{X, VIIa-TF}$ | 40 min^-1^, 390 nM | [8] |
| $k_{eff}^{X, VIIa}$ | 0.01 nM^-2^min^-1^ | [10]* |
| $k_{cat}^{X, IXa local}$, $K_{M}^{X, IXa local}/k$ | 0.6 min^-1^, 230 molecules/platelet | [12,13]* |
| $k_{cat}^{X, IXa-VIIIa local}$,  $K_{M}^{X, IXa-VIIIa local}/k$,  $K_{d}^{IXa-VIIIa local}/k$, $K_{d}^{VIIIa-X local}/k$ | 6350 min^-1^,  1216 molecules/platelet,  278 molecules/platelet,  1655 molecules/platelet | [14] |
| $k_{eff LP }^{II, Xa}$ | 0.002 nM^-1^min^-1^ | [15] |
| $\frac{k_{cat}^{local II, Xa-Va}}{K_{M}^{local II, Xa-Va}/k}$ | 0.047 min^-1^ | [16]* |
| $k_{cat}^{Fg, IIa}$, $K_{M}^{Fg, IIa}$ | 5040 min^-1^, 7200 nM | [17] |
| 3. Cofactor activation | | |
| $k_{cat}^{VIII, IIa}$, $K_{M}^{VIII, IIa}$ | 54 min^-1^, 147 nM | [18] |
| $k_{cat}^{V, IIa}$, $K_{M}^{V, IIa}$ | 14 min^-1^, 71.7 nM | [19] |
| 4. Inhibition | | |
| $k_{a}^{VIIa-TF, Xa-TFPI}$ | 0.44 nM^-1^min^-1^ | [20] |
| $k_{a}^{Xa-VIIa-TF, TFPI}$ | 6 nM^-1^min^-1^ | [9] |
| $k_{a}^{Xa, TFPI}$, $k_{d}^{Xa-TFPI}$ | 0.052 nM^-1^min^-1^, 0.02 min^-1^ | [20] |
| $k_{a}^{IXa, AT-III}$ | 0.0000082 nM^-1^min^-1^ | [21] |
| $k_{a}^{Xa, AT-III}$ | 0.00015 nM^-1^min^-1^ | [22] |
| $k_{a}^{Xa, \alpha_{2}M}$ | 0.00004 nM^-1^min^-1^ | [23] |
| $k_{a}^{Xa, \alpha_{1}AT}$ | 0.0000136 nM^-1^min^-1^ | [23] |
| $k_{a}^{Xa, PCI}$ | 0.0012 nM^-1^min^-1^ | [24] |
| $k_{a}^{Xa-Va^{B}, AT-III}$ | 0.000022 nM^-1^min^-1^ | [25] |
| $k_{a}^{IIa, AT-III}$ | 0.00041 nM^-1^min^-1^ | [22] |
| $k_{a}^{IIa, \alpha_{2}M}$ | 0.0001 nM^-1^min^-1^ | [26] |
| $k_{a}^{IIa, \alpha_{1}AT}$ | 0.000003 nM^-1^min^-1^ | [27] |
| $k_{a}^{IIa, PCI}$ | 0.00037 nM^-1^min^-1^ | [24] |
| $k_{a}^{IIa, HC-II}$ | 0.000063 nM^-1^min^-1^ | [28] |
| $k_{a}^{XIa, AT-III}$ | 0.000019 nM^-1^min^-1^ | [29] |
| $k_{a}^{XIa, \alpha_{2}AP}$ | 0.000026 nM^-1^min^-1^ | [29] |
| $k_{a}^{XIa, \alpha_{1}AT}$ | 0.000006 nM^-1^min^-1^ | [29] |
| $k_{a}^{XIa, PCI}$ | 0.0054 nM^-1^min^-1^ | [24] |
| $k_{a}^{XIa, C1I}$ | 0.00014 nM^-1^min^-1^ | [30] |
| $k_{a}^{PCa, \alpha_{2}M}$ | 0.000006 nM^-1^min^-1^ | [31] |
| $k_{a}^{PCa, \alpha_{2}AP}$ | 0.000006 nM^-1^min^-1^ | [31] |
| $k_{a}^{PCa, \alpha_{1}AT}$ | 0.0000007 nM^-1^min^-1^ | [27] |
| $k_{a}^{PCa, PCI}$ | 0.00039 nM^-1^min^-1^ | [24] |
| $h^{VIIIa}$ | 0.35 min^-1^ | [32] |
| $\frac{k_{cat}^{local Va, PCa}}{K_{M}^{local Va, PCa}}$ | 7.7 nM^-1^min^-1^ | [33]* |
| $\frac{k_{cat}^{local Va-PS, PCa}}{K_{M}^{local Va-PS, PCa}}$ | 12.6 nM^-1^min^-1^ | [33] |
| $k_{eff}^{PC, PCa}$ | 0.000282 nM^-1^min^-1^ | [34] |
| $K_{i}^{Va, PS}$ | 200 nM | [35]* |
| $K_{i}^{VIIIa, PS}$ | 150 nM | [36] |
| $k_{cat}^{PC, IIa}$, $K_{M}^{PC, IIa}$ | 1.2 min^-1^, 60000 nM | [37] |
| 5. Long-range feedback | | |
| $k_{eff}^{XI, IIa}$ | 0.03 nM^-2^min^-1^ | [38]* |
| 6. Phospholipids reactions | | |
| $K_{d}^{Xa-Va}$ | 0.118 nM | [16] |
| $n^{Va}$, $K_{d}^{Va}$ | 2700 sites/platelet, 2.9 nM | [16,39] |
| $n^{IXa}$, $K_{d}^{IXa}$ | 260 sites/platelet, 2.57 nM | [40] |
| $n^{VIIIa}$, $K_{d}^{VIIIa}$ | 750 sites/platelet, 1.5 nM | [41] |
| $n^{X(II)}$, $K_{d}^{X}$, $K_{d}^{II}$ | 16000 sites/platelet, 320 nM, 470 nM | [42] |
| $n^{XIa}$, $K_{d}^{XIa}$ | 250 sites/platelet, 1.7 nM | [43] |

* Estimated on the basis of experimental data.

Table S3. Model parameters: diffusion coefficients

| Factor | Diffusion coefficient*, mm^2^/min |
| --- | --- |
| VIIa | 0.0035 |
| VII | 0.0035 |
| IXa | 0.0037 |
| IX | 0.0033 |
| Xa | 0.0037 |
| X | 0.0033 |
| IIa | 0.0040 |
| II | 0.0030 |
| Fn | 0^¶^ |
| Fg | 0.0012 |
| VIIIa | 0.0021 |
| VIII | 0^#^ |
| Va | 0.0022 |
| V | 0.0016 |
| PCa | 0.0032 |
| PC | 0.0032 |
| Xa–TFPI | 0.0027 |
| TFPI | 0.0039 |
| AT-III | 0.0033 |
| XIa | 0.0021 |
| XI | 0.0021 |

* The values of diffusion coefficients were estimated on the basis of molecular weights of the components, using data from [44]

¶ Fibrin quickly polymerizes into fibrin net, and was therefore assumed not to diffuse.

# Factor VIII circulates in blood bound to von Willebrand factor, which exists in the form of huge complexes. We assumed these complexes not to diffuse.

## Numeric methods

### Solving Navier-Stokes Equations

*The grid.* In the quasistationary approach, alteration of the velocity field occurs after the clot has changed its form. To find the new velocity field, we use the iterative process described below.

The stationary Navier-Stokes equations are solved with the method described in [45]. Three noncoincident grids are used to calculate pressure and two components of flow velocity. Grid of cells with signed nodes of velocity grids and pressure grid is shown in Fig. 1.


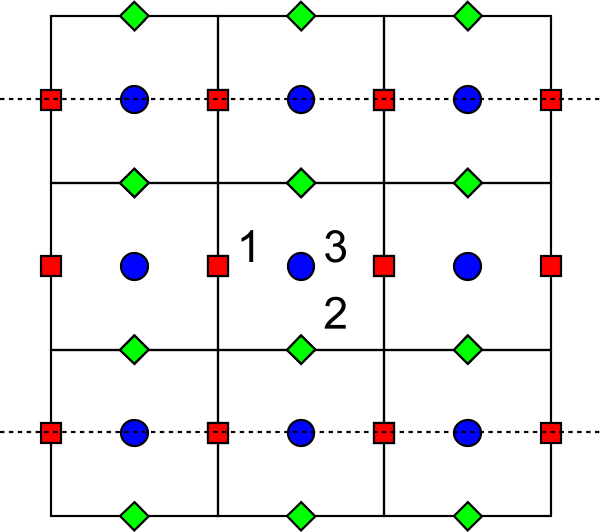


**Fig. 1 Mutually spaced grids for solution of Navier-Stokes equations.** The numbers indicate nodes of the three grids as follows: V_x_ value is calculated in nodes of grid 2; V_y_ value is calculated in the nodes of grid 1; P value and chemical reagent concentration value are calculated in the nodes of grid 3. The border of simulation area is shown as a dotted line and passes through the nodes of grids 1 and 3.

*Approximation of linear components.* The following boundary problem (here and below superscript *h* indicates the difference analogue of an operator):

$\begin{matrix} -\Delta^{h}u^{h}+\nabla^{h}p^{h}=0 \\ div^{h}u=0 \end{matrix}$ S68

where $u$ and $p$ are velocity and pressure, can be written as the following equations for implicit two-layer iterative process:

S69

Here, the following designations are used: , where *τ* is an iteration parameter, β is a parameter depending on the geometry of simulation area, В is some operator; $v$ and $q$ are some elements of the functional spaces of $u^{h}$ and $p^{h}$, respectively.

,

after substituting of these equations in the first equation of (S69), we receive:

$\left(B-\tau\Delta^{h}-\frac{\tau}{\beta}\nabla^{h}div^{h}\right)v_{t}=\Delta^{h}v+\frac{1}{\beta}\nabla^{h}div^{h}v-\nabla^{h}q$ S70

We choose $B-\tau\Delta^{h}-\frac{\tau}{\beta}\nabla^{h}div^{h}$= С, and the formulae for calculation will be as follows:

$\begin{matrix} C\frac{v^{n+1}-v^{n}}{\tau}=\Delta^{h}v^{n}+\frac{1}{\beta}\nabla^{h}div^{h}v^{n}-\nabla^{h}q^{n}, \\ q^{n+1}=q^{n}-\frac{1}{\beta}div^{h}v^{n+1} \end{matrix}$ S71

We construct discrete analog of operators and *div* as in [45], and choose operator C= *-∆^h^*.

*Approximation of nonlinear components.*

Construction of N(u^h^, v^h^) and approximation of nonlinear components $u_{k}v_{x_{k}}$ are as described in [45]. We define $v_{x}=u,v_{y}=v$; approximation of nonlinear components is as follows:

$$\begin{matrix} N_{x}(u,v)=\frac{1}{4\cdot h\cdot\nu}\cdot((u_{i,j}+u_{i-1,j})\cdot(u_{i,j}-u_{i-1,j})+(u_{i+1,j}+u_{i,j})\cdot(u_{i+1,j}-u_{i,j})+ \\ +(v_{i,j}+v_{i+1,j})\cdot(u_{i,j+1}-u_{i,j})+(v_{i,j-1}+v_{i+1,j-1})\cdot(u_{i.j}-u_{i,j-1})) \end{matrix}$$

S72

$$\begin{matrix} N_{y}(u,v)=\frac{1}{4\cdot h\cdot\nu}\cdot((v_{i,j}+v_{i,j-1})\cdot(v_{i,j}-v_{i,j-1})+(v_{i,j+1}+v_{i,j})\cdot(v_{i,j=1}-v_{i,j})+ \\ +(u_{i-1,j}+u_{i-1,j-1})\cdot(v_{i,j}-v_{i-1,j})+(u_{i,j}+u_{i,j+1})\cdot(v_{i+1.j}-v_{i,j})) \end{matrix}$$

So, the following finite-difference problem corresponds to the system (S68)

$\begin{matrix} -\nu\Delta^{h}\mathbf{u}^{h}+\nabla^{h}p+N(\mathbf{u}^{h},\mathbf{u}^{h})=0, \\ div\mathbf{u}^{h}=0 \end{matrix}$ S73

Obtained grid equations are solved using alternately-triangular method [46]. As an iteration parameter τ we use the Chebyshev set of iteration parameters $\tau_{k}$, ordered so that the scheme was stable [46]. Also, it reduced influence of approximation errors. $\tau_{k}$ is calculated in the following way:

$$\begin{matrix} \tau_{k}=\frac{\tau_{0}}{1+\rho_{0}\mu_{k}},\mu_{k}\in\aleph_{n}=\left\{\cos\frac{\Theta_{n}(i)\pi}{2n},1\leq i\leq n\right\},1\leq k\leq n, \\ \tau_{0}=\frac{2}{\gamma_{1}+\gamma_{2}}, \\ \rho_{0}=\frac{1-\xi}{1+\xi}, \\ \xi=\frac{\gamma_{1}}{\gamma_{2}} \end{matrix}$$

S74

S75

S76

S77

S78

S79

S80

S81

S82

We choose n=32. In order to regularize set $\aleph_{n}$ we construct the sequences of odd numbers $\Theta_{n}=\left\{\Theta_{n}(1),\Theta_{n}(2),...,\Theta_{n}(n)\right\},1\leq\Theta_{n}(i)\leq2n-1$, *i=1,2,…n.* Algorithm of the set $\Theta_{n}$ construction is based on step-by-step transition from set $\Theta_{m}$ to set $\Theta_{2m}$. Transition from $\Theta_{m}$ to $\Theta_{2m}$ is the following:

$\begin{matrix} \Theta_{2m}(2i-1)=\Theta_{m}(i), \\ \Theta_{2m}(2i)=4m-\Theta_{2m}(2i-1),i=1,2,...m \end{matrix}$ S83

As $\Theta_{1}=\left\{1\right\}$, for n=32 we receive

$$\begin{matrix} \Theta_{2}=\left\{1,3\right\}, \\ \Theta_{4}=\left\{1,7,3,5\right\}, \\ \Theta_{8}=\left\{1,15,7,9,3,13,5,11\right\}, \\ \Theta_{16}=\left\{1,31,15,17,7,25,9,23,3,29,13,19,5,27,11,21\right\}, \\ \Theta_{32}=\left\{1,63,31,33,15,49,17,47,7,57,25,39,9,55,23,41,3,61,29,35,13,51,19,45,5,59,27,37,11,53,21,43\right\} \end{matrix}$$

For equation $Ay=f$, where *A* =∆ and $f=-\frac{1}{\nu h}\left(\nabla q\right)+\frac{1}{\beta h}\left(\nabla divv\right)-N\left(v,v\right)$, iteration scheme is as follows:

$B\frac{y_{k+1}-y_{k}}{\tau_{k+1}}+Ay_{k}=f$, S84

where

$B=\left(D+\omega R_{1}\right)D^{-1}\left(D+\omega R_{2}\right)$ S85

We define *ω=ω_0_*, *D=E*, а *R_1_+R_2_=∆*, and using the following algorithm

S86

which can be presented in the following form:

S87

Here the following notations are used

$$\begin{matrix} \alpha=\frac{\omega_{0}}{h^{2}+2\omega_{0}}, \\ \beta=\frac{\omega_{0}}{h^{2}+2\omega_{0}}, \\ \chi=\frac{h^{2}}{h^{2}+2\omega_{0}}, \\ \omega_{0}=\frac{2}{\sqrt{\delta\Delta}} \end{matrix}$$

S88

S89

S90

S91

Within the area occupied by the clot, we assume $\omega(i,j)=\bar{\omega}(i,j)=0$.

So, we receive solution on the step k+1 on the basis of solution on step k. Each time, when we need to find out new flow velocities, we solve equations S87. We choose the previous values of flow velocities as initial condition (step 0), and continue these iterations until we have the steady solution ($divy<0.01$).

### Solving Biochemical Equations

We use method of alternating directions [46] to solve reaction-diffusion-convection equations. Iterative schemes for two adjacent steps have different forms:

$\begin{matrix} \frac{u_{ij}^{n+1}-u_{ij}^{n}}{\tau}=D\left(\frac{\partial^{2}u}{\partial x^{2}}\right)_{ij}^{n+1}+D\left(\frac{\partial^{2}u}{\partial y^{2}}\right)_{ij}^{n}-\left(V_{x}\left(\frac{\partial u}{\partial x}\right)^{n+1}\right)_{ij}-\left(V_{y}\left(\frac{\partial u}{\partial y}\right)^{n}\right)_{ij}+f(u^{n}), \\ \frac{u_{ij}^{n+2}-u_{ij}^{n+1}}{\tau}=D\left(\frac{\partial^{2}u}{\partial x^{2}}\right)_{ij}^{n+1}+D\left(\frac{\partial^{2}u}{\partial y^{2}}\right)_{ij}^{n+2}-\left(V_{x}\left(\frac{\partial u}{\partial x}\right)^{n+1}\right)_{ij}-\left(V_{y}\left(\frac{\partial u}{\partial y}\right)^{n+2}\right)_{ij}+f(u^{n}) \end{matrix}$ S92

where *u* is concentration of reagent, *D* is diffusion coefficient, *V* is flow velocity, *f* is chemical reaction. This iterative scheme provides a system of three-point difference equations for each step, for the first equation

$\begin{matrix} c_{0j}u_{0j}-b_{0j}u_{1j}=f_{0j},1\leq j\leq N_{x}-1, \\ -a_{ij}u_{i-1,j}+c_{ij}u_{ij}-b_{ij}u_{i+1,j}=f_{ij},1\leq i\leq N_{x}-1, \\ -a_{N_{x},j}u_{N_{x}-1,j}+c_{N_{x},j}u_{N_{x},j}=f_{N_{x},j} \end{matrix}$ S93

and for the second one

$\begin{matrix} c_{i0}u_{i0}-b_{i0}u_{i1}=f_{i0},1\leq i\leq N_{y}-1, \\ -a_{ij}u_{i,j-1}+c_{ij}u_{ij}-b_{ij}u_{i,j+1}=f_{ij},1\leq j\leq N_{y}-1, \\ -a_{i,N_{y}}u_{i,N_{y}-1}+c_{i,N_{y}}u_{i,N_{y}}=f_{i,N_{y}} \end{matrix}$ S94

Here *a*, *b*, *c* are coefficients.

We use the method of non-monotone marching [46] to solve these equations. It is based on the fact that matrixes of these equations could be without diagonal predominance:

$\begin{matrix} \left|c_{i}\right|\geq\left|a_{i}\right|+\left|b_{i}\right|,1\leq i\leq N-1, \\ \left|c_{0}\right|\geq\left|b_{0}\right|,\left|c_{N}\right|\geq\left|a_{N}\right| \end{matrix}$ S95

which is necessary for usage of monotone marching. Scheme of non-monotone marching has the following form (for the first scheme we choose j=const and omit this index; for the second scheme we do the same with index i):

$\begin{matrix} Cu_{m_{l}}-b_{l}u_{l+1}=F, \\ -Au_{m_{l}}+c_{l+1}u_{l+1}-b_{l+1}u_{l+2}=Ф, \\ -a_{l+2}u_{l+1}+c_{l+2}u_{l+2}-b_{l+2}u_{l+3}=f_{l+2}, \\ -a_{i}u_{i-1}+c_{i}u_{i}-b_{i}u_{i+1}=f_{i},l+3\leq i\leq N-1, \\ -a_{N}u_{N-1}+c_{N}u_{N}=f_{N}, \end{matrix}$ S96

where *m_l_*≤*l*.

Here θ and χ are two arrays of indexes:

$\theta=\left\{\theta_{i},1\leq i\leq N\right\},\chi=\left\{\chi_{i},1\leq i\leq N\right\}$ S97

When *l=0* *С=с_0_, A=a_1_, F=f_0_, Ф=f_1_, m_0_=0.* We put χ_0_=0. For *i=0,..N-1* cases 1 or 2 are carried out.

1. if $\left|C\right|\geq\left|b_{i}\right|$ then

$\begin{matrix} \alpha_{i+1}=\frac{b_{i}}{C}, \\ \beta_{i+1}=\frac{F}{C}, \\ C=c_{i+1}-A\alpha_{i+1}, \\ F=Ф+A\beta_{i+1}, \\ \theta_{i+1}=\chi_{i}, \\ \chi_{i+1}=i+1, \\ A=a_{i+2}, \\ Ф=f_{i+2} \end{matrix}$ S98

1. if$\left|C\right|$<$\left|b_{i}\right|$ then

$\begin{matrix} \alpha_{i+1}=\frac{C}{b_{i}}, \\ \beta_{i+1}=-\frac{F}{b_{i}}, \\ C=c_{i+1}\alpha_{i+1}-A, \\ F=Ф-c_{i+1}\beta_{i+1}, \\ \theta_{i+1}=i+1, \\ \chi_{i+1}=\chi_{i}, \\ A=a_{i+2}\alpha_{i+1}, \\ Ф=f_{i+2}+a_{i+2}\beta_{i+1} \end{matrix}$ S99

Then *u_n_* is calculated (here *n=χ_N_*) using formula *u_n_=F/C*. Then for *i=N-1, N-2,…,0* were calculated $u_{m}=\alpha_{i+1}u_{n}+\beta_{i+1},m=\theta_{i+1},n=\chi_{i+1}.$

We use the following scheme to approximate convective components.

$\left(V_{x}\left(\frac{\partial u}{\partial x}\right)^{n}\right)_{ij}=\frac{1}{h}\cdot V_{x}^{ij}(u_{ij}^{n}-u_{i-1,j}^{n})$, if $V_{x}^{ij}>0,V_{x}^{i+1,j}>0;$ S100

$\left(V_{x}\left(\frac{\partial u}{\partial x}\right)^{n}\right)_{ij}=\frac{1}{h}\cdot V_{x}^{i+1,j}(u_{i+1,j}^{n}-u_{i,j}^{n})$, if $V_{x}^{ij}<0,V_{x}^{i+1,j}<0;$ S101

$\left(V_{x}\left(\frac{\partial u}{\partial x}\right)^{n}\right)_{ij}=\frac{1}{h}\cdot\left(\frac{V_{x}^{ij}(u_{ij}^{n}-u_{i-1,j}^{n})}{2}+\frac{V_{x}^{i+1,j}(u_{i+1,j}^{n}-u_{ij}^{n})}{2}\right)$, if $V_{x}^{ij}\cdot V_{x}^{i+1,j}<0;$ S102

Approximation scheme for diffusion components is as follows:

$D\left(\frac{\partial^{2}u}{\partial x^{2}}\right)_{i,j}^{n}=D\cdot\frac{u_{i-1,j}^{n}-2\cdot u_{i,j}^{n}+u_{i+1,j}^{n}}{h^{2}}$ S103

We use the following iterative parameters, $h=\frac{Lx}{N_{x}}=\frac{Ly}{N_{y}}$*=0.01*; $\tau=0.01$.

### Estimation of Calculation Errors

Using numerical methods for solving equations, we obtain an approximate solution that differs from the exact. Here we use several approaches to test accuracy of this approximation.

The solution accuracy usually increases if the grid step decreases; dependence of the selected parameter of comparison (the integral of fibrin in the direction perpendicular to the activator) on the number of nodes per mm has an asymptote value (Fig. 2).

Fig.2 The dependence of the integral of fibrin in the direction perpendicular to the activator on the number of nodes per mm; the 2^nd^ minute of coagulation. Flow shear rate is 10s^-1^.

From this figure we conclude that the error of the solution using a grid with the parameter h = 0.01 mm is not more than 10%. Further reduction of spatial step leads to a substantial increase in the time of calculation (reduction of spatial step twice leads to the almost tenfold increase in the time of calculation), and does not appear necessary.

However, there is a calculation error due the choice of approximation method of convective components. It causes appearing of approximation viscosity [47]. Equation of reaction-diffusion-convection type

$\frac{\partial u}{\partial t}=D\frac{\partial^{2}u}{\partial x^{2}}+D\frac{\partial^{2}u}{\partial y^{2}}+V_{x}\frac{\partial u}{\partial x}+V_{y}\frac{\partial u}{\partial y}+f(u)$ S104

where *u* is concentration of reagent, *D* is diffusion coefficient, *V_x_* and *V_y_* are x and y components of flow velocity, *f(u)* is chemical reaction, has the following difference form:

S105

where $\tau$ and *h* are time and spatial grid steps.

The appearance of members of the form $ku''_{xx}$ is equivalent to increasing the diffusion coefficient $D_{eff}=D+D'(V,h,\tau)$, which causes additional smearing. As a first approximation, the increase of the diffusion coefficient is $D'\sim h\sqrt{V_{x}^{2}+V_{y}^{2}}$.

This value can be rather high under conditions of our study. However, the region of interest is the low-flow-rate region near the wall, where all biochemical reactions take place. Let us examine the value of approximation viscosity in this region.

A key element of the coagulation system is thrombin, which activates fibrinogen, which then forms a clot. An examination reveals that almost all thrombin is located inside the clot (Fig. 3), which protects it from the flow and increase of the diffusion coefficient due to the used approximation affects only a small concentration of thrombin outside the clot.

Fig.3 Profiles of the concentrations of fibrin, thrombin and the flow velocity in the direction perpendicular to the activator. Initial flow shear rate is 10 s^-1^, 14^th^ minute of calculation. Nearly all thrombin is inside the clot and is protected from the flow.

Thus thrombin is concentrated in the area of low flow velocities, for which the following equality $h\sqrt{V_{x}^{2}+V_{y}^{2}}\sim10^{-3}\sim D_{thrombin}$ is correct, i.e. contribution of the approximation viscosity is comparable with the contribution of diffusion. Assessment of the contribution of diffusion of thrombin in the initial process of forming a clot can be as follows: an increase in the diffusion coefficient of thrombin in 10 times leads to a two-fold increase in the time delay of clot appearance. So approximation viscosity contributes about 20% error in the timing of the appearance of the clot.

So, as the effects of flow influence on blood coagulations obtained in our calculations sufficiently exceed calculating error, we conclude that the findings of this paper are not influenced by the possible inaccuracies of the numeric method.

## Reference List

1. Panteleev MA, Ovanesov MV, Kireev DA, Shibeko AM, Sinauridze EI, Ananyeva NM, Butylin AA, Saenko EL, Ataullakhanov FI: **Spatial propagation and localization of blood coagulation are regulated by intrinsic and protein C pathways, respectively.** *Biophys J* 2006, **90:**1489-1500.

2. Batchelor GK: *Introduction To Fluid Dynamics, An.* Cambridge University Press; 2007.

3. Ovanesov MV, Krasotkina JV, Ul'yanova LI, Abushinova KV, Plyushch OP, Domogatskii SP, Vorob'ev AI, Ataullakhanov FI: **Hemophilia A and B are associated with abnormal spatial dynamics of clot growth.** *Biochim Biophys Acta* 2002, **1572:**45-57.

4. Rodgers GM, Broze GJ, Jr., Shuman MA: **The number of receptors for factor VII correlates with the ability of cultured cells to initiate coagulation.** *Blood* 1984, **63:**434-438.

5. Nemerson Y, Gentry R: **An ordered addition, essential activation model of the tissue factor pathway of coagulation: evidence for a conformational cage.** *Biochemistry* 1986, **25:**4020-4033.

6. Butenas S, Mann KG: **Kinetics of human factor VII activation.** *Biochemistry* 1996, **35:**1904-1910.

7. Rao LV, Williams T, Rapaport SI: **Studies of the activation of factor VII bound to tissue factor.** *Blood* 1996, **87:**3738-3748.

8. Komiyama Y, Pedersen AH, Kisiel W: **Proteolytic activation of human factors IX and X by recombinant human factor VIIa: effects of calcium, phospholipids, and tissue factor.** *Biochemistry* 1990, **29:**9418-9425.

9. Panteleev MA, Zarnitsina VI, Ataullakhanov FI: **Tissue factor pathway inhibitor: a possible mechanism of action.** *Eur J Biochem* 2002, **269:**2016-2031.

10. Monroe DM, Hoffman M, Oliver JA, Roberts HR: **Platelet activity of high-dose factor VIIa is independent of tissue factor.** *Br J Haematol* 1997, **99:**542-547.

11. Gailani D, Ho D, Sun MF, Cheng Q, Walsh PN: **Model for a factor IX activation complex on blood platelets: dimeric conformation of factor XIa is essential.** *Blood* 2001, **97:**3117-3122.

12. Scandura JM, Walsh PN: **Factor X bound to the surface of activated human platelets is preferentially activated by platelet-bound factor IXa.** *Biochemistry* 1996, **35:**8903-8913.

13. Rawala-Sheikh R, Ahmad SS, Ashby B, Walsh PN: **Kinetics of coagulation factor X activation by platelet-bound factor IXa.** *Biochemistry* 1990, **29:**2606-2611.

14. Panteleev MA, Saenko EL, Ananyeva NM, Ataullakhanov FI: **Kinetics of factor X activation by the membrane-bound complex of factor IXa and factor VIIIa.** *Biochem J* 2004, **381:**779-794.

15. Bajaj SP, Harmony JA, Martinez-Carrion M, Castellino FJ: **Human plasma lipoproteins as accelerators of prothrombin activation.** *J Biol Chem* 1976, **251:**5233-5236.

16. Tracy PB, Eide LL, Mann KG: **Human prothrombinase complex assembly and function on isolated peripheral blood cell populations.** *J Biol Chem* 1985, **260:**2119-2124.

17. Higgins DL, Lewis SD, Shafer JA: **Steady state kinetic parameters for the thrombin-catalyzed conversion of human fibrinogen to fibrin.** *J Biol Chem* 1983, **258:**9276-9282.

18. Hill-Eubanks DC, Lollar P: **von Willebrand factor is a cofactor for thrombin-catalyzed cleavage of the factor VIII light chain.** *J Biol Chem* 1990, **265:**17854-17858.

19. Monkovic DD, Tracy PB: **Activation of human factor V by factor Xa and thrombin.** *Biochemistry* 1990, **29:**1118-1128.

20. Baugh RJ, Broze GJ, Jr., Krishnaswamy S: **Regulation of extrinsic pathway factor Xa formation by tissue factor pathway inhibitor.** *J Biol Chem* 1998, **273:**4378-4386.

21. Pieters J, Willems G, Hemker HC, Lindhout T: **Inhibition of factor IXa and factor Xa by antithrombin III/heparin during factor X activation.** *J Biol Chem* 1988, **263:**15313-15318.

22. Rezaie AR: **Calcium enhances heparin catalysis of the antithrombin-factor Xa reaction by a template mechanism. Evidence that calcium alleviates Gla domain antagonism of heparin binding to factor Xa.** *J Biol Chem* 1998, **273:**16824-16827.

23. Ellis V, Scully M, MacGregor I, Kakkar V: **Inhibition of human factor Xa by various plasma protease inhibitors.** *Biochim Biophys Acta* 1982, **701:**24-31.

24. Espana F, Berrettini M, Griffin JH: **Purification and characterization of plasma protein C inhibitor.** *Thromb Res* 1989, **55:**369-384.

25. Ellis V, Scully MF, Kakkar VV: **Inhibition of prothrombinase complex by plasma proteinase inhibitors.** *Biochemistry* 1984, **23:**5882-5887.

26. Jesty J: **The kinetics of inhibition of alpha-thrombin in human plasma.** *J Biol Chem* 1986, **261:**10313-10318.

27. Heeb MJ, Bischoff R, Courtney M, Griffin JH: **Inhibition of activated protein C by recombinant alpha 1-antitrypsin variants with substitution of arginine or leucine for methionine358.** *J Biol Chem* 1990, **265:**2365-2369.

28. Derechin VM, Blinder MA, Tollefsen DM: **Substitution of arginine for Leu444 in the reactive site of heparin cofactor II enhances the rate of thrombin inhibition.** *J Biol Chem* 1990, **265:**5623-5628.

29. Wuillemin WA, Eldering E, Citarella F, de Ruig CP, ten Cate H, Hack CE: **Modulation of contact system proteases by glycosaminoglycans. Selective enhancement of the inhibition of factor XIa.** *J Biol Chem* 1996, **271:**12913-12918.

30. Meijers JC, Vlooswijk RA, Bouma BN: **Inhibition of human blood coagulation factor XIa by C-1 inhibitor.** *Biochemistry* 1988, **27:**959-963.

31. Heeb MJ, Gruber A, Griffin JH: **Identification of divalent metal ion-dependent inhibition of activated protein C by alpha 2-macroglobulin and alpha 2-antiplasmin in blood and comparisons to inhibition of factor Xa, thrombin, and plasmin.** *J Biol Chem* 1991, **266:**17606-17612.

32. Lollar P, Parker ET, Fay PJ: **Coagulant properties of hybrid human/porcine factor VIII molecules.** *J Biol Chem* 1992, **267:**23652-23657.

33. Solymoss S, Tucker MM, Tracy PB: **Kinetics of inactivation of membrane-bound factor Va by activated protein C. Protein S modulates factor Xa protection.** *J Biol Chem* 1988, **263:**14884-14890.

34. Hassouna H, Quinn C: **Proteolysis of protein C in pooled normal plasma and purified protein C by activated protein C (APC).** *Biophys Chem* 2002, **95:**109-124.

35. Hackeng TM, van ', V, Meijers JC, Bouma BN: **Human protein S inhibits prothrombinase complex activity on endothelial cells and platelets via direct interactions with factors Va and Xa.** *J Biol Chem* 1994, **269:**21051-21058.

36. Koppelman SJ, Hackeng TM, Sixma JJ, Bouma BN: **Inhibition of the intrinsic factor X activating complex by protein S: evidence for a specific binding of protein S to factor VIII.** *Blood* 1995, **86:**1062-1071.

37. Esmon NL, DeBault LE, Esmon CT: **Proteolytic formation and properties of gamma-carboxyglutamic acid-domainless protein C.** *J Biol Chem* 1983, **258:**5548-5553.

38. Oliver JA, Monroe DM, Roberts HR, Hoffman M: **Thrombin activates factor XI on activated platelets in the absence of factor XII.** *Arterioscler Thromb Vasc Biol* 1999, **19:**170-177.

39. Tracy PB, Nesheim ME, Mann KG: **Platelet factor Xa receptor.** *Methods Enzymol* 1992, **215:**329-360.

40. Ahmad SS, Rawala-Sheikh R, Walsh PN: **Comparative interactions of factor IX and factor IXa with human platelets.** *J Biol Chem* 1989, **264:**3244-3251.

41. Ahmad SS, Scandura JM, Walsh PN: **Structural and functional characterization of platelet receptor-mediated factor VIII binding.** *J Biol Chem* 2000, **275:**13071-13081.

42. Scandura JM, Ahmad SS, Walsh PN: **A binding site expressed on the surface of activated human platelets is shared by factor X and prothrombin.** *Biochemistry* 1996, **35:**8890-8902.

43. Baird TR, Walsh PN: **The interaction of factor XIa with activated platelets but not endothelial cells promotes the activation of factor IX in the consolidation phase of blood coagulation.** *J Biol Chem* 2002, **277:**38462-38467.

44. Marshal AG: *Biological chemistry: principles, technics, and applications.* New York: John Wiley and Sons; 1978.

45. Kobelkov GM: **On Numerical Methods of Solving the Navier-Stokes Equations in Velocity-Pressure Variables.** In *Numerical Methods and Applications* . Edited by Edited by Marchuk GI. CRC Press; 1994.

46. Samarskii AA, Nikolaev ES: *Numerical Methods for Grid Equations.* Birkhauser; 1989.

47. Kiryanov DV, Kiryanova EN: *Computational Science (Mathematics).* Jones and Bartlett Publishers; 2006.
