# Supplementary figures and images for: Blood flow controls coagulation onset via the positive feedback of factor VII activation by factor Xa
Source: BMC Syst Biol. 2010 Jan 26;4:5. doi: 10.1186/1752-0509-4-5 (PMC2823678; doi:10.1186/1752-0509-4-5)

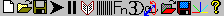

Supplement: Additional file 4 — Program code files. Source files of program realization of blood coagulation modeling under flow conditions. [file 1752-0509-4-5-S4.ZIP › Program code files/res/Toolbar.bmp]
