# Supplementary material for: Blood flow controls coagulation onset via the positive feedback of factor VII activation by factor Xa
Source: BMC Syst Biol. 2010 Jan 26;4:5. doi: 10.1186/1752-0509-4-5 (PMC2823678; doi:10.1186/1752-0509-4-5)
Supplement: Additional file 4 — Program code files. Source files of program realization of blood coagulation modeling under flow conditions. [file 1752-0509-4-5-S4.ZIP › Program code files ReadMe.doc]

Program code files folder contains source files of our program for simulation of blood coagulation under flow conditions.

You will need Microsoft Visual Studio 2005/2008 to make an executable file and run this program.

To do this, open file Flux2d.sln in Visual Studio workspace and then choose Debug->Start without debugging. The following window will appear.

To change model parameters, press Properties.

To change saving or viewing parameters, press Options.

To start calculations, press button 1 (with arrow). A folder with current date will appear in the folder with source files. It will contain a folder with current time, wherein all calculated data will be stored.

To process saved data, press button 2, then open the first file (e.g. 0.flx) with open dialog (button 3) and then press button 4. The file Fn3.profil contains data on fibrin concentration at different times in the direction perpendicular to the activator patch in the middle of the activator.

For any questions regarding this program please contact Alexey M. Shibeko via e-mail: alshibeko@gmail.com
